# *DICER1* mutation and pituitary prolactinoma

**DOI:** 10.1530/EDM-18-0087

**Published:** 2018-10-01

**Authors:** Ellena Cotton, David Ray

**Affiliations:** 1 Faculty of Biology, Medicine and Health, University of Manchester, Manchester Academic Health Sciences Centre, Manchester, UK; 2 Specialist Medicine, Manchester University Foundation Trust, Manchester, UK; 3 Oxford Centre for Diabetes, Endocrinology and Metabolism, University of Oxford, Oxford, UK

## Abstract

A young woman carrying germline *DICER1* mutation was discovered to have a pituitary microprolactinoma when she became amenorrhoic. The mutation was identified as a result of family screening following the early death of the patient’s daughter with ovarian cancer. The patient was in follow-up screening for thyroid disease, and investigations were initiated when she became amenorrhoic. MR scan revealed a 6 mm diameter pituitary microadenoma and raised prolactin. The prolactin was efficiently suppressed with low-dose cabergoline, and her menstrual cycles resumed. Dicer is an RNase enzyme, which is essential for processing small non-coding RNAs. These molecules play pleiotropic roles in regulating gene expression, by targeting mRNA sequences for degradation. *DICER1* plays different roles depending on cell context, but is thought to be a functional tumour suppressor gene. Accordingly, germline mutation in one *DICER1* allele is insufficient for oncogenesis, and a second hit on the other allele is required, as a result of postnatal somatic mutation. Loss of *DICER1* is linked to multiple tumours, with prominent endocrine representation. Multinodular goitre is frequent, with increased risk of differentiated thyroid cancer. Rare, developmental pituitary tumours are reported, including pituitary blastoma, but not reports of functional pituitary adenomas. As *DICER1* mutations are rare, case reports are the only means to identify new manifestations and to inform appropriate screening protocols.

**Learning points:**

- *DICER1* mutations lead to endocrine tumours.
- *DICER1* is required for small non-coding RNA expression.
- *DICER1* carriage and microprolactinoma are both rare, but here are reported in the same individual, suggesting association.
- Endocrine follow-up of patients carrying *DICER1* mutations should consider pituitary disease.

## Background

Prolactinomas are the most common type of pituitary tumour. Most prolactinomas are sporadic, but genetic syndromes increase risk. The aim of this report is to present a female patient, carrying a *DICER1* mutation, who developed a pituitary prolactinoma. The tumour was responsive to cabergoline therapy.

## Case presentation

**Figure 1:**
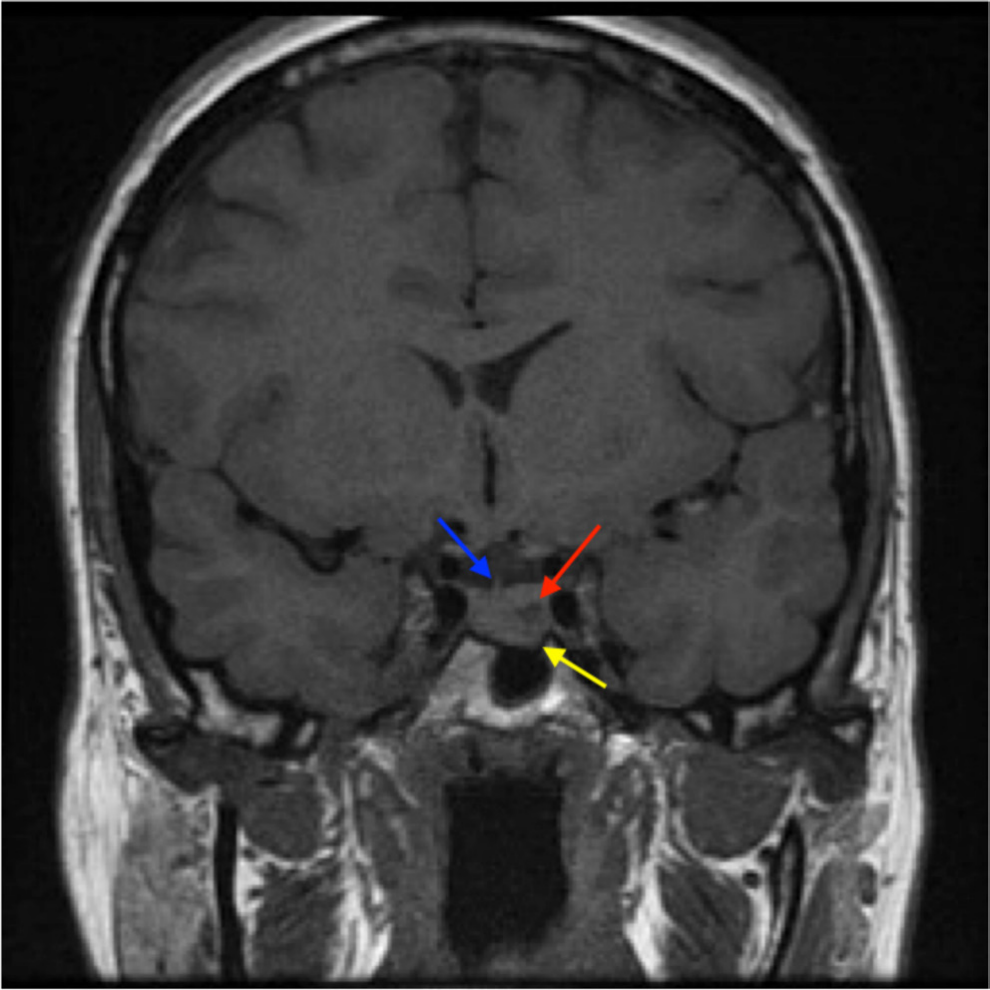
MR Scan in January 2010. Coronal T1-weighted MR scan revealing a mass within the pituitary gland on the left (red arrow). The pituitary stalk is deviated to the right (blue arrow) as a result and the floor of the pituitary gland is sloping down to the left hand side (yellow arrow) eroding into the pituitary fossa. These findings are entirely consistent with a pituitary microadenoma.

A 50-year-old woman presented, in 2010, with galactorrhoea and oligomenorrhea of 4 years. Her past medical history included a right nephrectomy following a road traffic accident as a child. She had five children. The galactorrhea persisted since the birth of her last son when she was 31 years old. Endocrinology assessment revealed raised serum prolactin at 1444 mu/L with suppressed gonadotrophins and oestradiol. The patient was not taking any medication at the time. Renal function and thyroid function were both normal. A pituitary MR scan showed a 6 mm diameter microadenoma with inferior extension towards the sphenoid sinus on the left hand side (Fig. 1). The patient also suffered from headaches and visual disturbance, although the tumour was confined within the sella turcica and was not thought to be causal. Taken together, these findings were interpreted as diagnostic of a microprolactinoma.

After the patient’s eldest daughter died in her early 20s in 2013 from ovarian cancer, genetic testing was carried out within the family. The patient, as well as the patient’s mother and half-sister were found to carry a *DICER1* gene mutation. There is no other significant family history of pituitary disorders. As part of *DICER1* follow-up, she had regular scans of her thyroid, which revealed the expected multinodular goitre, and in 2018, she was diagnosed with a differentiated papillary carcinoma thyroid.

## Investigation

Endocrinology assessment revealed raised serum prolactin at 1444 mu/L with suppressed gonadotrophins and oestradiol. The normal range for prolactin levels in non-pregnant females is 106–850 mlU/L. A pituitary MR scan showed a microadenoma with inferior extension towards the sphenoid sinus on the left hand side.

**Figure 2:**
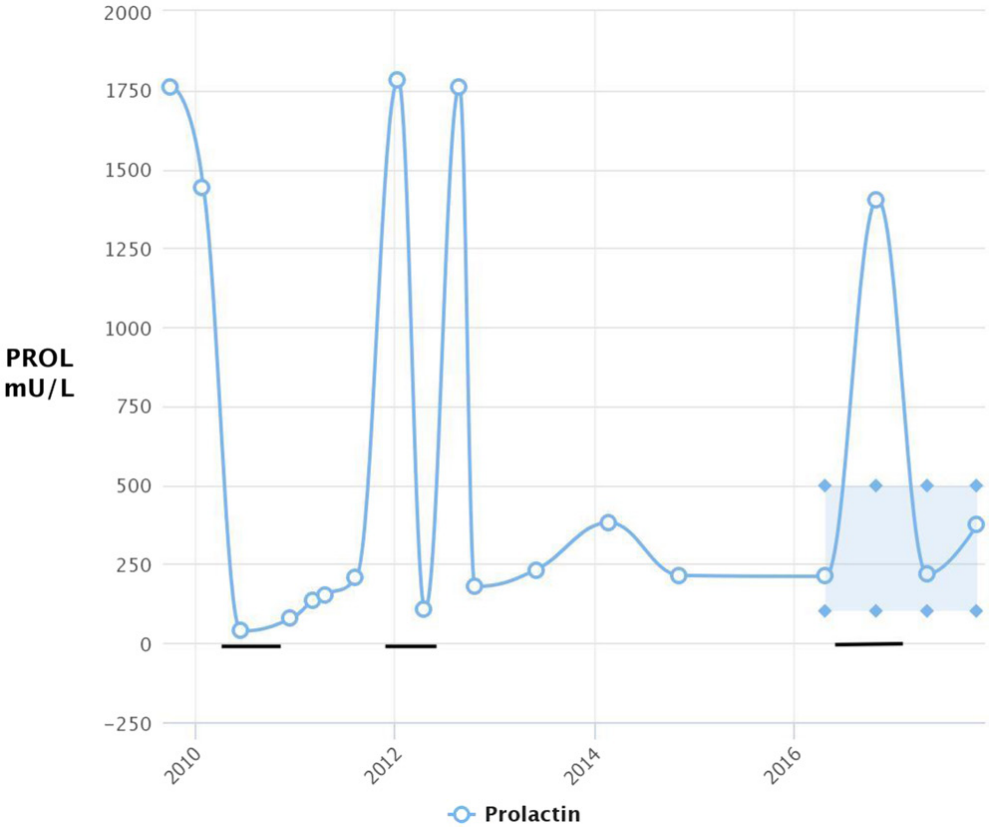
Line graph to show patient’s prolactin levels between 2010 and 2016. The blue shaded area indicates the normal range in a population for prolactin levels. The normal range for non-pregnant females is around 106–850 mlU/L. The black horizontal lines indicate when cabergoline treatment was commenced.

## Treatment

Following diagnosis, the patient was treated with cabergoline (250 µg once weekly orally, with a view in mind to raise the dose, aiming to reduce the size of the prolactinoma. The patient was on cabergoline for 69 months in total. The patient changed to quinagolide (75 µg daily) for 14 months between 2012 and 2013 but stopped and recommenced cabergoline (Fig. 2). The patient was on topiramate for 8 months between 2012 and 2013 for basilar migraines, but this medication has been stopped.

## Outcome and follow-up

Review in clinic 3 months after diagnosis showed the patient had continued to take cabergoline (500 µg once weekly, reduced to 250 µg in April 2018) and was no longer suffering from oligomenorrhea but still had persistent headaches. A repeat MR scan was conducted 6 months after initial diagnosis showing no reduction in the height of the left side of the pituitary, measuring 6 mm. A further scan in 2012 showed no significant change. The left-sided pituitary adenoma remained, but there was no interval growth.

The lack of tumour regression raises the possibility that this is a non-functioning pituitary adenoma, causing hyperprolactinaemia through stalk disruption.

## Discussion

This is a rare presentation of a patient who has developed a prolactinoma on the background of carrying a *DICER1* mutation. The cause of prolactinomas is mainly sporadic; however, in some cases, the cause can be genetic. In this case report, the patient carries a *DICER1* mutation, and this could possibly explain the cause of the prolactinoma they are being treated for. The link between these two rare occurrences has not yet been reported, and it could be argued that the two are connected.

miRNAs are small non-coding RNAs that regulate the expression of genes post transcriptionally (1). The underlying mechanisms of these miRNA remain unclear and the role they play in pituitary development and growth is not yet well understood (1). DICER1 encodes an enzyme, which is important for the processing of non-coding RNA species into microRNAs, and therefore, has pleiotropic functions.

A study from Zhang *et al*. (1) looked into the function of miRNA in the anterior pituitary by deleting DICER1. The study showed that dicer1 mutant mice had a loss of mature miRNA, and this led to hypoplasia in the pituitary and multiple hormone deficiencies (1). A further study used human tumour data from The Cancer Genome Atlas to profile the effects of DICER1 mutations on the miRNA profiles of the patients (2). The study showed that biallelic DICER1 mutations cause a change in the miRNA structure, which may contribute to oncogenesis (2).

A review from Tang *et al.* (3) suggests that experimental knockout of DICER1 leads to DNA damage (1). In this work, it was proposed that the efficiency of DNA damage repair was reduced in cells with mutations in DICER10-like genes such as *dcl2* (3). When DNA becomes damaged, the normal response is to begin a repair process. If this repair process is faulty, the residual damaged DNA can contribute to oncogenesis (3). This study tested this hypothesis by knocking out DICER1 in human cells, which resulted in accumulation of DNA breaks (3).

A study of prostate cancer by Bian *et al.* (4) suggests that down regulation of DICER1 is linked to cell proliferation and apoptosis. Furthermore, the expression of DICER1 was negatively associated with Gleason scoring (4). The prostate cancer data suggest a role for DICER1 as a tumour-suppressor gene, and we now report a patient with a DICER1 mutation who also suffers from a prolactinoma.

De Kock *et al.* (5) published a study concluding that loss-of-heterozygosity germline *DICER1* mutations contribute to the development of an aggressive and rare cancer called a pituitary blastoma (PitB) (5). While this was not looking into prolactinomas, the pathogenesis of cancer is similar and it demonstrates that the presence of a *DICER1* mutation can contribute to pituitary tumorigenesis. The results from the study suggest that PitB should be considered a ‘rare but pathognomonic’^(p8)^ (5) manifestation of a *DICER1* germline mutation (5). Coupled with this, the study also suggested that a somatic *DICER1* mutation in the RNase IIIb domain also appears to play a key role in the PitB pathogenesis (5). Despite its small sample size of 12, this study strongly proposes the link between *DICER1* mutations and the development of a pituitary tumour (5). In one case, there was an RNase IIIb missense mutation in the tumour sample, suggesting again this loss-of-function mutation than can lead to carcinogenesis (5).

Caimari *et al.* (6) also suggest that in more than 50 reported *DICER1* mutations, germline mutations of this kind often lead to truncated proteins (6). The study also suggests there can be a ‘second hit’ somatic mutation in the catalytic RNase IIIa and b domains (6). However, the mechanisms underlying this remain unclear. The study also states that the reasons behind *DICER1* germline mutations leading to tumorigenesis remain unclear and more research needs to be conducted in this area (6). Likewise, a study from Aksoy *et al.* (2) also observed that there is a second disabling genomic event in *DICER1* RNase III mutant samples and this mutation also affects the other *DICER1* allele. In essence, mutations in *DICER1* are biallelic and a second genomic mutation or ‘hit’ to the enzyme can contribute to oncogenesis (2). This suggests that these germline mutations in *DICER1* have been associated with cancer (2).

A study by Brenneman *et al.* (7) observed that in the case of pleuropulmonary blastoma (PPB), the majority of germline mutations were truncating loss-of-function mutations in a cohort of 124 PPB patients (90 out of 124 showed this kind of mutation) (7). The majority of these truncating mutations are single nucleotide substitutions that produce new stop codons and small deletions within exons that cause a shift in the reading frame (7). Of the 90 samples that showed this loss-of-function mutation, 84 showed germline *DICER1* mutations that truncate the open reading frame before the end of the important RNaseIIIb domain (7). This has been predicted to result in a complete loss of *DICER1* function (7). This study strongly suggests that the *DICER1* loss-of-function mutation can be enough to promote tumorigenesis (7).

This report has demonstrated compelling evidence that people who carry the *DICER1* mutations are at an increased risk of developing tumours. In particular, and in this one case, a prolactinoma. It is thought that truncating germline mutations or focused somatic missense mutations are unique to *DICER1* (8). Their relationship may be facilitated by miRNAs or a ‘yet-to-be identified’ (8) mechanism that affects the dicer protein (8).

One area of interest is the role of *DICER1* in DNA repair (3). Mutations, mostly leading to loss of expression, have been found in endometrial, ovarian and colon cancer amongst many others (9). In addition, hereditary* DICER1* loss-of-function mutations have so far been associated with pleuropulmonary blastoma (7). The mechanisms linking loss of dicer to cancer remain unclear, but may relate to DNA damage, which if defective can lead to accumulation of damaging mutations, cell transformation and potentially cell proliferation and carcinogenesis.

The emphasis remains on improving the care for patients who carry *DICER1*, in particular, screening for tumours that are linked to the mutation. To our knowledge, this case is the first report of prolactinoma in a patient with* DICER1*. Therefore, the question must be asked as to whether these two occurrences are linked, which suggests the possibility of *DICER1* mutations contributing to the development of prolactinomas.

The prevalence of* DICER1* mutations in the general population is 1 in 10,600 (10). There are two large cohort studies involving individuals with *DICER1:* ‘International PBB registry for PBB, *DICER1* and Associated Conditions’ and the NIH cohort study Natural History of *DICER1*. It will be useful to track pituitary pathology in these cohorts, to further strengthen the causal link with prolactinoma. This report should be considered a good study to build from previous reports of a similar nature and should hopefully help pave the way towards ubiquitous connections between *DICER1* and prolactinomas and ultimately, better treatment and outcomes for patients.

## Declaration of interest

The authors declare that there is no conflict of interest that could be perceived as prejudicing the impartiality of the research reported.

## Funding

D Ray is a Wellcome Investigator.

## Patient consent

Signed, informed consent has been obtained from the patient for the publication of this report.

## Author contribution statement

D Ray was the clinician caring for the patient. E Cotton was a medical student attached to the firm.
